# Oligodendrocyte lineage cells dysfunction in depression: early life stress, adolescent vulnerability and the emerging role of lipid metabolism

**DOI:** 10.1038/s41398-025-03765-x

**Published:** 2025-11-22

**Authors:** Chenyu Gao, Mengyu Liu, Jude Uzoechina, Zhijun Zhang

**Affiliations:** 1 Department of Neurology in Affiliated Zhongda Hospital and Jiangsu Provincial Medical Key Discipline, School of Medicine, Institute of Neuropsychiatry, Key Laboratory of Developmental Genes and Human Disease, Southeast University, Nanjing, China; 2 Shenzhen Key Laboratory of Precision Diagnosis and Treatment of Depression, Department of Mental Health and Public Health, Faculty of Life and Health Sciences of Shenzhen University of Advanced Technology, Shenzhen, China; 3 The Brain Cognition and Brain Disease institute, Shenzhen Institutes of Advanced Technology, Chinese Academy of Sciences, Shenzhen, China; 4 University of Chinese Academy of Sciences, Beijing, China

**Keywords:** Psychology, Neuroscience

## Abstract

Depression, as a serious global public health issue, is exhibiting an increasing incidence among younger populations, particularly adolescents, who face unique diagnostic challenges and poorer prognoses. Despite extensive studies on monoaminergic dysfunction, neuroinflammation, and synaptic deficits, its pathophysiological mechanisms remain incompletely understood, particularly in relation to developmental stage-specific vulnerabilities. Oligodendrocyte (OL) lineage cells have recently emerged as potential contributors to depression pathology, not only through their myelinating roles but also via non-myelinating functions, such as metabolic support, neuroimmune interaction, and circuit modulation. Early life represents a critical development window characterized by rapid proliferation, differentiation, and lipid synthesis of oligodendrocyte precursor cells, during which these cells are highly susceptible to environmental stressors. Such developmental susceptibility may underlie the long-lasting impact of early life stress (ELS) contribute to depression risk across the lifespan. This review summarizes recent advances in understanding the myelinating and non-myelinating functions of OL lineage cells related to depression pathology, with particular emphasis on their developmental vulnerability to ELS and potential contribution of lipid metabolic dysregulation. We further review emerging pharmacological and non-pharmacological strategies targeting OL lineage cells as potential therapeutic methods.

## Introduction

According to the latest World Health Organization report, approximately 280 million individuals worldwide are affected by depression, with rising incidence and a trend toward younger onset [1, 2]. Adolescents represent one of the fastest-growing depression populations, demonstrating significantly higher suicide risks compared to adult patients [2–5]. While core symptoms of depression include low mood, diminished interest, and anhedonia, adolescent patients more often present with irritability and frequent somatic complaints (headache, abdominal pain, etc.) [3, 6]. These atypical presentations leads to misdiagnosis and worse prognosis [6, 7].

The pathogenesis of depression remains incompletely understood. Multiple hypotheses have been proposed and extensively investigated, including the monoamine hypothesis, impaired synaptic plasticity, chronic low-grade inflammation causing peripheral-to-central inflammatory cascades, hypothalamic-pituitary-adrenal axis hyperactivation, and microglia-driven neuroinflammation [8–11]. While these hypotheses provide valuable insights into depression pathology, most derive from adult animal models and thus overlook the unique vulnerabilities of the developing brain.

Oligodendrocyte (OL) lineage cells, from oligodendrocyte precursor cells (OPCs) to immature oligodendrocytes (OLs) and mature OLs, are essential for brain function. Mature OLs are well known for their role in myelin sheath formation and metabolic support of axons [12, 13], while OPCs also regulate neural development, angiogenesis, and neural circuit plasticity, as well as influencing inflammation [14].During early life period, rapid proliferation and differentiation make these cells highly plastic yet vulnerable to stress [15], which can produce amplified and long-lasting effects on neural circuit maturation and brain function.

Emerging evidences have linked OL lineage cells dysfunction not only to white matter abnormalities but also to disruptions in metabolic support, immune crosstalk, and neuroplasticity network regulation that were relevant to depression [14, 16–18]. Recent single-nucleus transcriptomic profiling revealed substantial transcriptional dysregulation in OPCs in the dorsolateral prefrontal cortex of male individuals with depression [19], further implicating OL lineage cells as molecular contributors. Although most mechanistic insights come from adults, these findings nonetheless provide a valuable framework for exploring similar effects during sensitive developmental periods.

Myelin is composed of over 70% lipids, making lipid metabolism critical for OLs function and myelin maintenance [20]. Recent researches have highlighted altered lipid metabolism in depression [21–27], however, direct evidence linking OLs lipid dysfunction to pathology of depression remains limited. Given the critical role of lipid homeostasis in OLs biology and myelin integrity, lipid metabolism dysregulation represents a promising, yet underexplored, pathway through which OL lineage cells may contribute to the pathology of depression.

This review summarizes current evidence on myelinating and non-myelinating functions of OL lineage cells and their involvement in lipid metabolic dysregulation, with a focus on how early life stress (ELS) may confer heightened vulnerability to depression. We also discuss therapeutic strategies targeting OL lineage cells.

## The developmental origins and functions of OL lineage cells

Understanding the developmental origins of OL lineage cells is essential for contextualizing their functional vulnerabilities in neuropsychiatric disorders. The spatial-temporal pattern of the generation of OPCs shapes cellular heterogeneity and region-specific myelination programs, potentially influencing the susceptibility of myelinated circuits in both white and gray matter to early life or adolescent insults.

The developmental origins of the OL lineage cells have been well characterized in rodents in previous reviews [28–31]. In rodents, OL lineage cells arise in temporally staggered waves from ventral forebrain source (medial ganglionic eminence [MGE] / anterior entopeduncular area, ~E12.5; lateral ganglionic eminence [LGE] / the caudal ganglionic eminence [CGE], ~E15.5) and from dorsal cortex postnatally, with newer lineage analyses indicating that MGE-derived cells persist into adulthood whereas LGE/CGE contributions to adult cortex were limited [32–38]. In humans development occurs within an expanded outer subventricular zone rich in outer radial glia (oRG) [39–43]; although early study suggested that oRG could directly generate EGFR^+^ pre-OPCs [44], more recent single-cell and lineage-tracing data indicated that OPCs were primarily derived from EGFR^+^ basal multipotent intermediate progenitor cells produced by truncated radial glia in the ventricular zone rather than from oRG [45]. These conserved and primate-specific ontogenetic routes establish the initial distribution and molecular diversity of OL lineage cells. Although the majority of OPCs are specified during embryonic development, they retain a remarkable capacity for proliferation and differentiation throughout life [15, 46–48]. This lifelong plasticity peaks during adolescence, when myelination programs are still expanding and neural circuits remain highly malleable [46, 49].

**Fig. 1:**
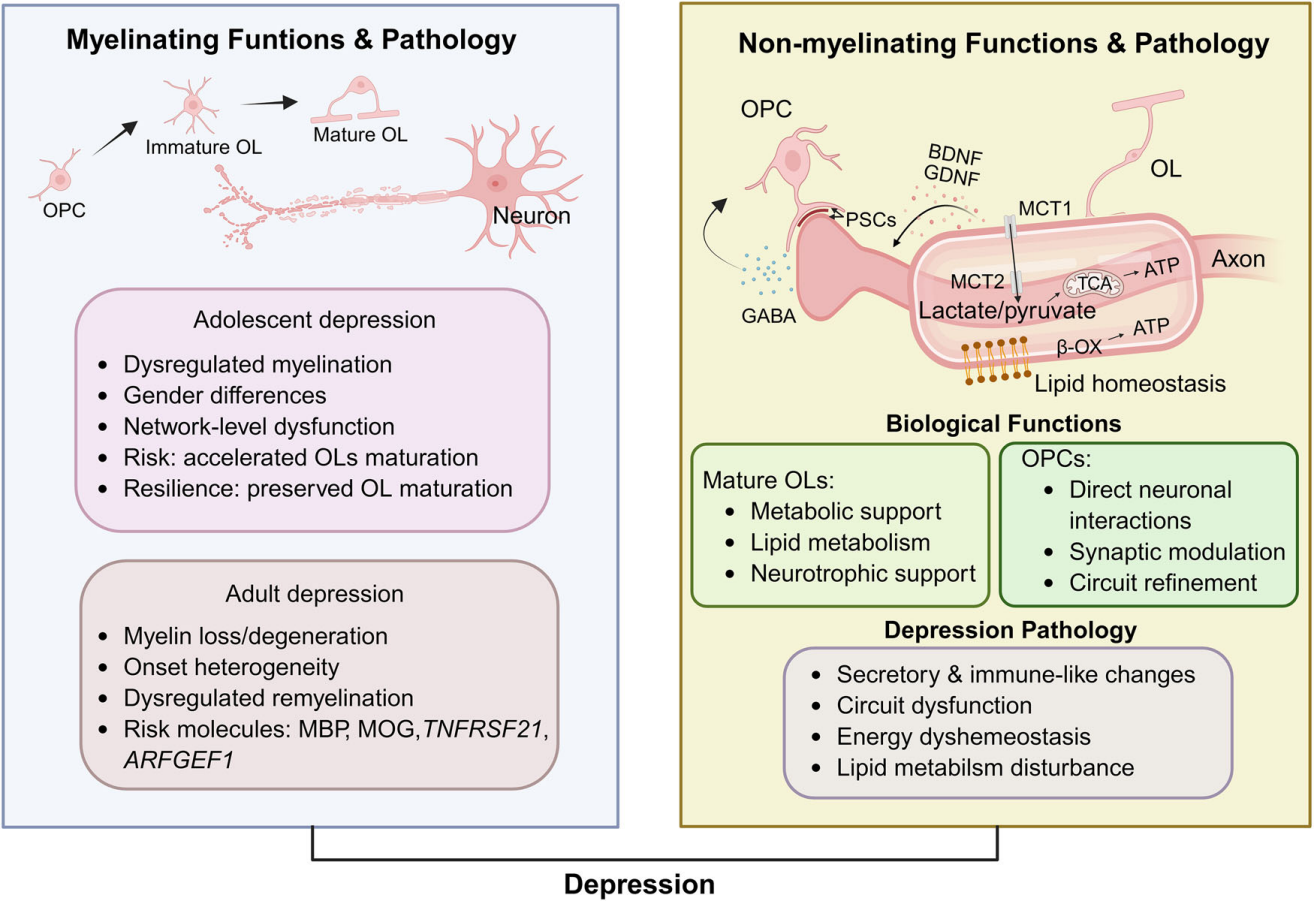
An overview of OL lineage cells dysfunction in depression pathology. OL lineage cells exhibits both myelinating functions and non-myelinating functions, and their pathological alternations contribute to depression. In adolescents, pathology includes dysregulated myelination, network-level dysfunctions, and sex-specific vulnerabilities, whereas adult pathology features progressive myelin loss, impaired remyelination, and molecular risk signatures (e.g., MBP, MOG, *TNFRSF21*, *ARFGEF1*). Beyond myelination, mature OLs provide metabolic support to axons by shuttling glycolysis-derived lactate/pyruvate and engaging lipids β-oxidation (β-OX) under low glucose condition, while secreting neurotrophic factors (BDNF, GDNF). OPCs interact with neurons via PSCs, modulate synaptic activity, and refine circuits. Non-myelinating pathology in depression includes secretory and immune-like changes, circuit dysfunction, energy dyshomeostasis, and lipid metabolism disturbance. Abbreviation: TCA, tricarboxylic acid cycle. Created in BioRender. Gao, C. (2025) https://BioRender.com/728czh4.

Across the lifespan, OL lineage cells fulfill distinct stage-specific functions. Specifically, mature OLs primarily form and maintain compact myelin sheaths around axons [50]. Paranode, specialized structures adjacent to nodes of Ranvier, anchor myelin lamellae via cell adhesion molecule networks to ensure the precision of saltatory conduction [51]. Beyond this myelinating role, mature OLs contribute to axonal support and neural circuit stability through multiple non-myelinating functions, including metabolic and alternative energy support via monocarboxylic acid transporters (MCTs)-mediated lactate/pyruvate shuttling during glucose deprivation [13, 52, 53], lipid metabolism for membrane integrity [54, 55], and secretion of neurotrophic factors such as brain-derived neurotrophic factor (BDNF) and glial cell line-derived neurotrophic factor (GDNF) to promote synaptic stability, neuronal survival, and axonal growth [56–58] (Fig. 1). OPCs retain life-long proliferation and differentiation capacity to generate new OLs, rapidly responding to neuronal activity to support adaptive myelination and maintain the plasticity of learning and emotion related circuits [12]. Beyond this myelinating potential, OPCs participate in diverse non-myelinating functions, including direct process-somata contacts (PSCs) with neurons to modulate lysosomal activity and metabolism in activity dependent manner [17], forming synapse-like structures with interneurons to release γ-aminobutyric acid (GABA) and regulate the excitation-inhibition balance [59], and engaging in axon engulfment and synaptic pruning to refine neural circuits [60–62] (Fig. 1).

These developmental and functional attributes, particularly the extended plasticity of OPCs and the structural specialization of mature OLs, make OL lineage cells indispensable for circuit maturation while also rendering them sensitive to perturbations during adolescence, when myelination trajectories and stress responsiveness converge, creating a heightened window of vulnerability for neural network stability.

## OL lineage cells dysfunction in depression pathology

A growing body of evidence has indicated that OL lineage cells are actively involved in the pathology of depression [63]. Structural and molecular disruptions in mature OLs, affecting myelin architecture, and in OPCs, affecting supportive and regulatory functions, have been consistently linked to reduced neural network efficiency and altered stress responsivity [64–68]. Cross-species and multi-scale investigations, including in vivo neuroimaging, human postmortem analyses, animal model pathology, and multi-omics profiling, have converged on a central theme: white matter microstructural abnormalities, myelin impairment, and oligodendrocyte lineage dysfunction are recurrent features of depression [64, 66, 69–74].

### OLs pathology in depression

Multimodal neuroimaging, postmortem, and mechanistic studies have indicated that mature OLs are disrupted in depression, with distinct signatures in adolescent versus adult cases (Fig. 1). In adolescents, diffusion tensor imaging (DTI) studies have consistently revealed altered white matter microstructure in emotion-regulatory tracts, including reduced fractional anisotropy (FA) and increased radial diffusivity (RD) in the uncinate fasciculus (UF), corpus callosum genu, corona radiata, and dorsal cingulum bundle, reflecting delayed or dysregulated myelination and disrupted connectivity in emotion regulatory networks [70, 75–79]. Ho et al. [70] further indicated sex-specific differences, with female adolescent patients showing increased R1, a myelin content proxy, in the UF and callosal genu; higher R1 in the left UF was also positively associated with current depression severity. This suggests that experience-driven increases in myelination may occur during episodes of depression in female adolescents, which could, when occurring during sensitive developmental periods, contribute to heightened vulnerability. In line with this, longitudinal and familial-risk studies have shown that trajectories of myelin maturation and white matter microstructure interact with psychosocial stressors, jointly shaping resilience or susceptibility to depression during adolescence [80–82].

In adults, depression is more often associated with loss or degeneration of previously established myelin. Multimodal imaging studies using DTI and myelin-sensitive quantitative MRI studies have consistently shown reduced white matter integrity in key tracts and regions, including the fornix, thalamus, inferior fronto-occipital fasciculus, uncinate fasciculus, orbitofrontal cortex, and anterior cingulate cortex (ACC), with the severity of structural impairment correlating with clinical symptom intensity [67, 71, 83–89]. Notably, patients with early-onset depression (defined variably as ≤25 or ≤ 30 years) showed increased FA value and mean RD value according to the DTI study along with an increased peripheral blood myelin oligodendrocyte glycoprotein (MOG) level, in contrast to the reduction observed in late-onset cases [75, 84]. Such findings point toward potential pathological heterogeneity across onset-age subtypes of depression and highlight the need for longitudinal and biomarker-based studies to clarify OL-related mechanisms in younger patients. Postmortem histopathology studies confirmed reduced OLs density and compact myelin integrity in the prefrontal cortex (PFC) and ACC, often accompanied by compensatory OPCs proliferation [64, 66]. Besides, a genome-wide study identified genetic variants in *TNFRSF21* (involved in OLs maturation) and *ARFGEF1* (involved in myelination) that were associated with cognitive deficits in depression [73]. Consistently, cross-species transcriptomic analysis identified conserved OLs dysregulation across diverse stress paradigms and in patients with depression, further consolidating OLs dysfunction as a core pathological feature [74].Chronic stress paradigms in adult rodents recapitulated these changes, producing shortened and thinned myelin sheaths in hippocampal and cortical regions such as medial prefrontal cortex (mPFC), as well as functional deficits in mature OLs [72, 90]; however, only reduced myelin protein concentration were observed in the nucleus accumbens (NAc), highlighting region-specific vulnerability [69]. Notably, chronic stress led to disrupted nodes of Ranvier, altered Caspr and Kv1.1 distribution, and reduced cAMP and membrane potential via OL-specific SGK1-mGluR signaling, impairing axonal conduction and associated behaviors [90]. Although astrocyte-derived lactate has been linked to neuronal excitability and depressive-like behaviors in rodents [91], there is currently no direct evidence that lactate derived from OL lineage cells modulates mood-related circuit. Nonetheless, OL lineage cells displayed metabolic heterogeneity, where OPCs and immature OLs produce lactate via higher lactate dehydrogenase (LDH) expression, while mature OLs mainly deliver pyruvate [92]. Whether developmental shifts in OL metabolic support contribute to adolescent vulnerability to depression remains unclear, and may warrant future investigation.

Beyond energy metabolism, OL-specific disruption of iron homeostasis was shown to trigger oxidative stress, neuroinflammation, and impaired synaptogenesis in PFC and hippocampus, driving depressive-like behavior in mice [93]. It underscored iron regulation as an additional non-myelinating vulnerability of OLs in depression beyond energy metabolism.

Together, these findings show that adolescent depression involves disrupted myelin maturation, whereas adult depression is marked by degeneration and functional impairment of mature OLs. Such distinctions have implications for timing and targeting of myelin-protective interventions.

### OPCs pathology in depression

Chronic stress consistently induces dynamic responses in OPCs, although the direction and magnitude of these changes vary across brain regions, stress paradigms, and developmental stages. In adult rodents, chronic social stress decreased proliferative OPCs in the mPFC while simultaneously increasing mature OLs in the amygdala, highlighting brain region-specific remodeling [94]. Similarly, chronic social defeat stress (CSDS) in susceptible mice elevated OPCs density yet reduced mature OLs in the mPFC, without altering total OPCs density, suggesting impaired differentiation rather than cell loss [69]. Genetic ablation of NG2^+^ OPC in the mPFC induced depressive like behavior, further implicating OPCs involved in depression through mechanisms such as reduced secretion of FGF2 [65]. Chronic stress paradigms, including CSDS or circadian misalignment, induced hypomyelination in the prefrontal cortex accompanied by OPCs morphological deficits, aberrant differentiation, and emergence of immune-like OLs (Im-OLs), which might integrate stress responses with myelin disruption [68, 95]. In adolescent mice, OPC-specific perturbations such as OL *ITPR2* deletion interrupted calcium homeostasis, suppressed proliferation, and reduced mature OLs, leading to depressive- and anxiety-like behaviors [96]. In humans, OPC-like progenitors from the olfactory epithelium correlated with cognitive performance and showed plastic responses to antidepressant treatment, suggesting peripheral OPCs populations may reflect or contribute to CNS mood-related processes [97]. Despite variations in proliferation and differentiation patterns, a common theme across these studies is that OPCs respond to stressors with lineage disruption, which contributes to depression pathology. The observed heterogeneity underscores the need for further investigation of brain region-, age-, and stress paradigm-specific OPCs alternations, particularly in adolescence, a period of heightened vulnerability.

Beyond their roles as progenitors, OPCs exerted direct regulatory functions on neuronal circuits that may contribute to depression pathology [17, 59, 98, 99] (Fig. 1). At the cellular level, OPCs could form bona fide synapses with glutamatergic and GABAergic neurons, dynamically modulating excitatory-inhibitory balance via activity-dependent calcium signaling and receptor expression, with disruptions linked to maladaptive circuit remodeling under stress [98, 100–102]. In addition to serving as recipients of neuronal input, OPCs could actively release neurotransmitters: selective photoactivation of NG2^+^ glia was shown to drive GABA release onto interneurons, enhancing inhibitory transmission and triggering anxiety-like behaviors that contribute to chronic social defeat stress phenotypes [59]. Moreover, it has been reported that OPCs are involved in synaptic pruning and axon engulfment, disturbing circuit refinement during critical developmental windows and promoting network rigidity [60–62, 103]. To date, there has been no direct evidence showing that chronic stress disrupts OPC-mediated synaptic pruning or axon engulfment. However, parallel evidence in microglia, where chronic social defeat stress drove complement-dependent excessive synaptic pruning and connectivity loss in the mPFC, suggesting that glia-mediated synaptic remodeling contributed to stress-related behavioral abnormalities [104, 105]. Given OPCs’ capacity for synaptic interactions and axonal remodeling, their potential involvement in stress-induced circuit rigidity remains an important and understudied field.

Together, these findings have suggested that OPCs dysfunction extends beyond lineage arrest to mechanistic bridge between stress, circuit dysregulation, and the emergence of depressive-like behaviors.

## Effects of ELS on OL lineage cells and myelin plasticity

The differentiation of OPCs into myelinating mature OLs persists throughout lifespan, with peak differentiation activity occurring during early childhood and stabilizing in adolescence [15, 49]. Beyond the congenital developmental program, myelin also undergoes experience-dependent and activity-driven adaptive development [106]. Current researches have demonstrated that newly generated mature OLs can participate in highly dynamic adaptive myelination processes not only in adulthood but also during early developmental stage [107, 108]. This process exhibited high sensitivity to external stimuli inputs, providing structural foundations for early-stage neural plasticity while simultaneously creating potential susceptibility for adolescent depression [15, 109].

### Early life stage: a critical window for OL lineage cells development

During early life stage, the peak periods of OPCs differentiation and myelination coincide with windows of neuron plasticity, suggesting potential contributions of developing OL-neuron crosstalk to depression pathology [110, 111]. During development, neurons dynamically communicated with OLs through synapse-like structures to precisely regulate myelination and its plasticity [17, 62, 112]. In adolescent mice, monocular visual deprivation delayed OPCs differentiation and maturation in the visual cortex; correspondingly, blocking OPCs differentiation reduced dendritic spine density, weakened inhibitory synaptic transmission, and induced enhanced plasticity in adulthood [113]. This indicates that early-life OPCs maturation critically stabilizes neural circuits and constrains neural plasticity during adulthood. Moreover, it has been reported that OPCs have similar synaptic pruning function as microglia during development stage, further demonstrating the importance of OLs in the regulation of neural plasticity during developmental stage [60–62]. The dynamics of lipid metabolism in OLs during early life stage is a central driver of myelin formation. OLs ensured efficient myelin formation and dynamic remodeling by precisely regulating the synthesis, storage, and reuse of cholesterol and sphingolipids [114]. In the brain, cholesterol synthesis peaks during adolescence and then enters a steady state, which is one of the phases of rapid myelin formation [115]. These collectively suggest that early life stage is a critical window for OL lineage cells development and function.

### Long-term effects of ELS

Accumulating evidences have indicated that ELS disrupted OLs development and myelination through multiple ways [116] (Table 1). A prospective cohort study of over 2000 adolescents demonstrated that family-environment stress (e.g., harsh parenting and domestic conflict) significantly impair cortical myelination in the ACC [117]; while a longitudinal neuroimaging finding revealed that childhood socioeconomic deprivation broadly decelerates myelination across cortical, subcortical, and core white matter regions [118]. Postmortem studies further identified OLs homeostasis disruption in the ACC and ventromedial prefrontal cortex (vmPFC) white matter of depression patients with childhood maltreatment histories, potentially linked to persistent myelination deficits mediated by epigenetic reprogramming [119, 120]. Consistently, a lipidomic postmortem analysis reported dysregulated fatty acid composition in the ACC myelin phospholipid pool of depressed suicides with child abuse histories, pointing to metabolic vulnerability of myelin membranes under ELS [121]. Another postmortem study of abused children showed upregulated expression of perineuronal nets (PNNs)-related genes (e.g., *VCAN, PTPRZ1, TNR*) in OPCs concomitant with increased cortical PNNs density, suggesting ELS may drive persistent affective disorders through OPCs-mediated PNNs malformation and subsequent neural plasticity impairment [122]. More recently, these findings have been extended to the basolateral amygdala (BLA) in another human postmortem study, where a novel nuclei-sorting method revealed reduced expression of the myelin-associated gene *MOBP* in OLs from depression patients with childhood maltreatment histories [123]. Notably, OPCs/OLs densities and myelin coverage were preserved, pointing instead to transcriptional dysregulation of OL lineage cells in limbic circuits as a potential mechanism linking ELS to depression [123]. A recent study using a neonatal hippocampus subfield MRI framework revealed delayed hippocampus myelination in preterm infants, with persistent deficit up to term-equivalent age despite preserved hippocampus volume [124]. This impairment showed reginal specificity, being more pronounced in the CA1 region and less affected in the CA2 region, potentially underlying the elevated long-term risk of depression [124].

**Table 1:** Long-term effects of ELS on OL lineage cells development and function.

Research type	Stress paradigm	Stress period	Assessment age	Core brain regions	OL lineage cells-associated changes	Ref
Prospective cohort study	Family-environment stress	Before 6 years of age	8-14 years of age	ACC	Myelin deficit	[117]
Human neuroimaging study	Childhood socioeconomic deprivation	Mostly before 12 years of age	Median age 18.7 years	Cortical, subcortical, and core white matter regions	Myelin deficit	[118]
Human neuroimaging study	Premature labor	Gestational age between 23- and 44-weeks	0.9-8.6 weeks after birth	Hippocampus	Delayed myelination	[124]
Postmortem study	Child abuse	Before 15 years of age	Mostly adult	ACC	OLs genes DNA methylation alternation Myelin deficit MBP ↓	[119]
Postmortem study	Child abuse	Before 15 years of age	Adult	vmPFC	Mature OLs↑ MAS1 ↑ Immature OLs ↓	[120]
Postmortem study	Child abuse	Before 15 years of age	Adult	vmPFC	OPCs-mediated PNNs formation ↓	[122]
Postmortem study	Child abuse	N/A	N/A	BLA	*MOBP*↓ in OLs	[123]
Postmortem study	Child abuse	Before 15 years of age	Adult	ACC	Delayed myelination, arachidonic acid synthesis dysregulation	[121]
Non-human primate study	MIA	1^st^ and 2^nd^ trimester	3.5–4 years of age	Hippocampus	*OLIG2, SOX10, MYRF, MBP, CNP, MOG, MAG* ↑ *PDGFRA* ↓	[138]
Non-human primate study	VFD	2-6 months of age	Around 5 years of age	Prefrontal limbic white matter region	Myelin deficit	[126]
Non-human primate study	VFD	2-6 months of age	Around 5 years of age	Anterior limb white matter region	Myelin deficit	[125]
Rodent study	MS	P2-14	P15 and adult	PFC	OPCs differentiation ↑ Myelin deficits	[128]
Rodent study	ELS (foot shock)	P21-26	P75-82	Hippocampus	OPCs differentiation ↓	[129]
Rodent study	CSDS	P28-37	P40	PFC, LHb	OPCs proliferation/differentiation ↓	[130]
Rodent study	CSDS	P28-37	P40	Amygdala, MHb	OPCs proliferation/differentiation ↑	[130]
Rodent study	Chronic social isolation	P21-65/ P21-35	P65	PFC	Myelin deficit OLs↓ MBP↓	[131]
Rodent study	MS	P2-12	P23	Hippocampus	OPCs ↓	[132]
Rodent study	MS	P2-21	P42	mPFC	A1R signaling pathway ↓ OPCs and OLs ↓	[133]
Rodent study	Acute juvenile traumatic stress	P28	P40 and P95	PFC, hippocampus, amygdala	Myelination alternations depend on sex	[134]
Rodent study	HFD for dams	Pregnancy and lactation	Adolescent and adult	PFC	MOG, MAL, CNPase ↓ OLs ↓	[135]
Rodent study	Cafeteria diet for dams	9 weeks (pre-pregnancy, pregnancy, and lactation)	P60	NAc, hippocampus, PFC	Decreased myelination	[136]
Rodent study	ELS of dams	P2-15	P21	BLA	*Mag, Mbp* ↓	[137]
Rodent study	Early social isolation of sires and offspring	P21-41 (sires); P21-34 (offspring)	P34	mPFC	MBP ↓ hypomyelination	[139]

Various animal studies have progressively elucidated ELS-induced OL lineage cells impairments. DTI studies in adult rhesus macaques depressive-like behavior models exposed to variable foraging demand stress (VFD) during early life have shown that such ELS selectively impairs prefrontal-limbic white matter integrity, manifesting as desynchronization of white matter maturation in anterior brain regions and aberrant compensatory coordination in posterior regions [125, 126]. A meta-analysis by Orso et al. [127] demonstrated that ELS or prenatal stress (PNS) exposure increased microglial density with somatic hypertrophy while reducing OLs density in rodent brains. Teissier et al. [128] revealed that maternal separation (MS) drived premature differentiation of OPCs in PFC of mice, resulting in OPCs pool depletion, myelination deficits, and subsequent depressive-like behaviors in adulthood. This process was mediated by abnormalities in neuron-glia interactions driven by inhibition of neuron activity in PFC [128]. In contrast, ELS-exposed rat models exhibited distinct OL lineage cells dysfunction characterized by impaired OPCs differentiation capacity, reduced mature OLs numbers, and enhanced apoptosis – all paralleled by elevated oxidative stress and persistent anxiety/depressive-like behavior [129]. Notably, the effect of ELS has been demonstrated spatiotemporal specificity: adolescent chronic stress selectively suppresses OPCs proliferation/differentiation in the PFC and lateral habenula (LHb), but spares the amygdala and medial habenula (MHb) [130]. Chronic social isolation lasting the entire adolescent period (postnatal days 21-65, P21-P65) induced simpler OLs morphology without any change of OLs density in the mPFC of mice [131]. Crucially, even short-term isolation during early adolescence (P21-P35) sufficed to induce similar pathological alterations, whereas identical stress exposure after P35 (approximately mid-to-late adolescent) produced no significant effects [131]. Mechanistic investigation revealed that juvenile social experience regulates the “critical time window” for maturation of OLs in the PFC through the ErbB3 signaling pathway, demonstrating precise spatiotemporal specificity - an effect absent in the motor cortex [131]. These findings collectively suggest that OL lineage cells developmental impairment exhibits strict spatial (brain-region-dependent) and temporal (developmental-stage-specific) constraints. Furthermore, MS was shown to reduce OPCs population in the hippocampus, inhibit Wnt7b paracrine signaling, thereby suppressing Wnt/β-catenin pathway-dependent astrocyte development, ultimately leading to depressive-like behaviors in mice [132]. An update study further demonstrated that MS activated peripheral CD4^+^ T cells and elevated xanthine levels, which inhibited the adenosine A1 receptor (A1R) signaling pathway of OPCs in the PFC, resulting in myelin damage and anxiety/depressive-like phenotypes [133], suggesting OPCs might serve as key mediators of peripheral immune-central nervous system crosstalk in depression pathogenesis. A single acute juvenile traumatic stress exposure induced sex-specific long-term alterations in gray matter myelination within PFC, amygdala and hippocampus in adulthood, with female rats exhibiting widespread myelin reduction whereas males showed no persistent effects [134]. In contrast, this stress increases gray matter myelination in the amygdala and hippocampus of adolescent male rats, while reduced the number of OLs in the PFC of adolescent female rats in the short term [134]. This indicated potential sexual heterogeneity in ELS-induced long-term and short-term consequences.

As an important part of early life stress, the influence of PNS on the development of OLs in offspring should not be ignored. A high-fat diet during pregnancy inhibited OLs maturation and suppressed myelination-related gene/protein expression in the PFC of the offspring, thereby inducing depressive-like behaviors that persist into adulthood [135]. Notably, this study specifically identified male-biased myelination-related protein deficits [135], which paradoxically contrasted with the female-specific myelination impairment caused by acute juvenile stress mentioned below [134]. This discrepancy suggests a stress-type-dependent sexual dimorphism in the regulation of OL lineage cells. Similarly, maternal exposure to a cafeteria diet during pregnancy and lactation primed depressive-like behavior in offspring, accompanied by reduced volume in the PFC, hippocampus, and NAc, as well as synaptic deficits and decreased myelination in the dentate gyrus [136]. Consistently, expression of myelination-related genes (e.g., *Mag, Mbp*) was reduced in the BLA of offspring whose mother experienced ELS, concurrent with DNA methylation elevated [137]. Critically, this effect demonstrated dynamic interactions with perinatal selective serotonin reuptake inhibitors (SSRIs) exposure, with male offspring exhibiting heightened sensitivity to such combined stressors [137]. Furthermore, in a maternal immune activation (MIA) non-human primate model, prenatal exposure to the viral mimetic poly(I:C) induced hippocampus-specific OL lineage cells abnormality in adolescent offsprings (developmentally equivalent to humans aged 14-16 years) [138]. The abnormality featured upregulated myelination-related genes (*OLIG2, SOX10, MYRF, MBP, CNP, MOG, MAG*) and downregulated OPCs marker gene *PDGFRA*, accompanied by increased social deficits and stereotypic behaviors [138]. It has also been reported that paternal early social isolation epigenetically elevates sperm miR-124, impairing maturation of OLs and myelination in the mPFC of offsprings, thereby disrupting social behavior across generations [139]. Collectively, these findings indicate that prenatal environmental stress reshape OL lineage cells developmental trajectories, thereby exerting transgenerational effects on offspring neural circuit function.

Taken together, evidence from human postmortem, neuroimaging, and animal models indicates that ELS persistent and multifaceted impacts on OL lineage cells development and myelination through transcriptional, epigenetic, immune, and neuronal activity-dependent regulation. Future work is needed to disentangle these interacting pathways and identify therapeutic windows for intervention.

## Dysregulation of lipid metabolism in OLs

OLs are highly active in lipid metabolism, which is indispensable for both myelination and broader central nervous system homeostasis. Myelin membranes are composed of over 70% lipids, with cholesterol and sphingolipids constituting the major fraction [20]. Beyond myelin synthesis, OLs dynamically exchange fatty acids and cholesterol with neurons and astrocytes to support energy metabolism, oxidative protection, and synaptic plasticity [20, 55, 140, 141]. Multiple clinical studies have demonstrated that lipid metabolism dysregulation was prevalent in both adult and adolescent depression [23, 26, 142], which has strong correlations with depression severity, cognitive deficits, and prognosis [25, 27, 143]. Thus, disruption of OLs lipid homeostasis probably represent a crucial nexus linking stress exposure to myelin deficits and depression.

### Dysregulation of cholesterol metabolism

During myelination, OLs synthesize cholesterol for membrane construction, and this biosynthetic activity persists in adulthood to maintain myelin stability and axonal support [114].Chronic stress has been shown to impair this process by suppressing phosphorylation of 3-hydroxy-3-methylglutaryl-CoA synthase (HMGCR), a rate-limiting enzyme in cholesterol biosynthesis, leading to reductions in myelin proteins such as MBP and MOG and consequent white matter disruption [22]. Single-nucleus RNA sequencing further demonstrated persistent downregulation of cholesterol transporters genes (e.g., *Apoe*, *Apod*) and regulatory factors genes (e.g., *Dhcr24*, *Srebf2*) in OLs in the hypothalamus of mice after chronic stress, resulting in abnormal cholesterol accumulation, altered OLs developmental trajectories, and depressive-like behaviors [24]. In patients with depression, downregulation of the myelin-related gene *CNP* exacerbates cholesterol metabolic dysfunction, which results in axonal metabolic support failure and synaptic plasticity impairment [144]. Such dysregulation interacts with chronic stress-induced neuroinflammation, forming a vicious cycle: pro-inflammatory factors (e.g., IL-1β, TNF-α) suppress cholesterol synthase expression, while abnormal cholesterol metabolites amplify microglia activation, collectively aggravating inflammation-mediated OPCs differentiation defects and myelin damage [145–147]. Significantly, the antidepressant venlafaxine could restore HMGCR activity to ameliorate cholesterol metabolism, suggesting therapeutic potential [22].

### Dysregulation of sphingolipid metabolism

Sphingolipids serve as essential lipids for OLs to maintain myelin structure and signaling functions, constituting 30–40% of myelin lipids [20]. Their metabolites, such as ceramides, act as bioactive mediators of inflammation responses under stress or injury conditions [20]. Accumulating evidence underscored the indispensable role of central sphingolipid metabolic dysregulation in depression pathology [21, 23, 148]. In animal models, stress exposure enhanced sphingomyelinase/ceramidase activity in emotion-related regions (e.g., hippocampus and PFC), correlating with depressive-like behaviors and impaired myelin integrity [148–150]. Direct evidence linking shingolipid metabolism to adolescent oligodendrocyte dysfunction in depression is currently lacking, representing an important knowledge gap for future research.

Together, these findings underscored OLs lipid homeostasis as an emerging mechanistic link between stress, myelination deficits, and depression. A recent study demeonstrated that ELS induced widespread lipid metabolism dysregulation in the depressed rat brain [151], supporting the notion that ELS broadly perturbs lipid homeostasis, although direct evidence linking these alternations to OL lineage cells remains limited. While most evidence centers on cholesterol pathways, and much of the current data treats lipid metabolism as a largely parallel pathway, this emerging evidence suggests that lipid dysregulation could represent a convergent mechanism mediating the effects of ELS on OL lineage cells development and myelination. Future studies, particularly in adolescent models, are needed to clarify whether and how dysregulation of OL lipid homeostasis contributes to ELS-induced OL dysfunction, and whether therapeutic modulation of these pathways can retore myelin integrity and resilience against depression.

## Therapeutic strategies targeting OL lineage cells

**Fig. 2:**
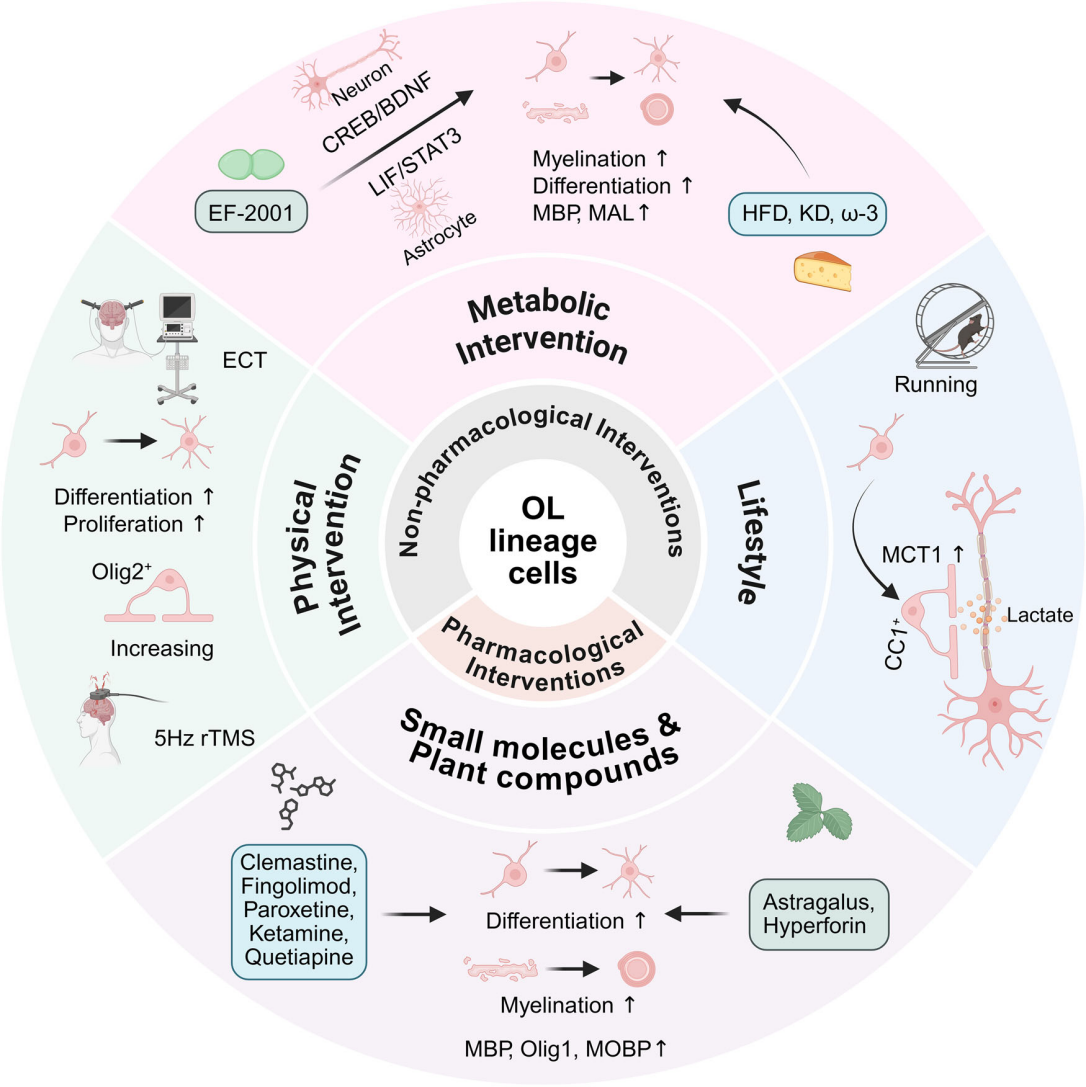
Therapeutic strategies targeting OL lineage cells in depression. Both pharmacological and non-pharmacological interventions have demonstrated potential in improving depression-related OL lineage cells dysfunction through multiple mechanisms. Small-molecule drugs such as clementine, fingolimod, paroxetine, ketamine, and quetiapine promote OPCs differentiation and enhance myelin repair. Plant compounds exert antidepressant effects by upregulating OL-specific transcription factors and improving mitochondrial and myelin function. Non-pharmacological interventions - including physical interventions, metabolic therapies, and lifestyle interventions - promote myelin plasticity, enhance glia-neuron crosstalk and metabolic coupling. Created in BioRender. Gao, C. (2025) https://BioRender.com/rt2cf2j.

In recent years, diverse interventions, ranging from small-molecule drugs to lifestyle modifications, have been shown to ameliorate or prevent depression-associated myelin damage through multiple mechanisms: promoting OPCs differentiation, enhancing myelin repair, remodeling lipid/energy metabolism, and alleviating inflammatory responses (Fig. 2). These findings provide a wealth of candidates for developing novel antidepressant strategies centered on OLs regulation.

### Pharmacological interventions

Clemastine, a classical antihistamine, has been demonstrated in vitro and in vivo to promote OPCs differentiation into mature OLs and accelerate remyelination in lysolecithin-induced demyelination models [152–154]. Its efficacy has been validated in a randomized, controlled, double-blind crossover clinical trial for multiple sclerosis [155]. Preclinical studies (including in adolescent social isolation models and CSDS models) have further revealed its capacity to rescue stress-induced depressive-like behaviors through enhanced OPCs differentiation, myelin repair, and neuroinflammatory modulation [156–160]. However, emerging evidence suggested that clemastine’s impact may be age- and context- deprendent: in developmental models, clemastine increased OPCs differentiation but paradoxically impaired myelin formation and conduction velocity, potentially via disruption of microglia-OL crosstalk [161].These findings highlight the need for caution when considering clemastine in pediatric or adolescent populations, and underscore the importance of microglia-OL interactions in mediating promyelinating therapies. Fingolimod (FTY720), a non-selective sphingosine-1-phosphate G protein-coupled receptor modulator, not only enhanced OPCs survival and migration but also accelerated myelin regeneration in chemical-induced demyelination models, concomitant with improved cognitive and emotional behaviors [162–165]. The classic SSRIs paroxetine was reported to exhibit antidepressant effects partially through promoting OPCs proliferation/differentiation and subsequent myelin repairment [166, 167]. Ketamine, a rapid-acting antidepressant drug, was recently shown to repair CSDS-induced myelin damage via α-amino-3-hydroxy-5-methyl-4-isoxazolepropionic acid receptor-mediated OPCs differentiation; suppression of myelin oligodendrocyte basic protein expression abolishes its sustained antidepressant effects [168]. Quetiapine, an atypical antipsychotic with established adjunctive efficacy in depression [169, 170], enhanced oligodendrocyte differentiation and myelination through dual mechanisms: downregulating Cdkn1a expression in OPCs/neurons and increasing nuclear-to-cytoplasmic translocation of Olig1 transcription factor [169, 171]. Plant-derived bioactive compounds have demonstrated significant therapeutic potential in various neuropsychiatric disorders, with growing recognition of its antidepressant properties. Recent research revealed that total flavonoids from Astragalus (a primary active constituent of Astragalus root) exerted antidepressant effects by enhancing hippocampus myelination in chronic unpredictable mild stress models [172]. This process is mediated through upregulation of OLs transcription factors Olig2/Sox10 and concurrent suppression of the Wnt/β-catenin signaling pathway [172]. *Hypericum perforatum* (St. John’s Wort), a well-established botanical antidepressant, showed emerging potential in adolescent depression management [173]. Its bioactive constituent, hyperforin, not only promoted OPCs differentiation but also enhanced mitochondrial function, while concurrently protecting against mitochondrial toxicity-induced damage [174]. These findings suggest a candidate mechanism through which *Hypericum perforatum* may exert antidepressant effects, at least in part, by supporting OL lineage cell function.

In summary, pharmacological strategies targeting OL lineage cells, including classical antidepressants and antipsychotics, remyelination-promoting agents, and plant-derived bioactive compounds, have demonstrated multifaceted therapeutic potential. These drugs exert their effects through promoting OPCs proliferation and differentiation, enhancing myelin repair, and modulating neuroinflammatory responses. Nevertheless, most of these pharmacological data originate from adult or preclinical studies, and their safety profiles in adolescent populations remain largely uncharacterized. Age-related differences in pharmacokinetics, off-target neurodevelopmental effects, and potential interference with ongoing myelination could pose additional risks. Recent studies in adolescents (e.g., Ketamine [175] and deep transcranial magnetic stimulation [TMS] [176]) have shown acceptable safety and tolerability, but long-term data on OL/myelin outcomes are still lacking. Thus, rigorous age-specific safety evaluations will be crucial before clinical translation of OL-targeting interventions in youth.

### Non-pharmacological interventions

Non-pharmacological antidepressant strategies targeting OL lineage cells are becoming a research hotspot. Electroconvulsive therapy (ECT) counteracts glucocorticoid-induced inhibition of OLs generation by restoring the proliferation of OPCs and promoting their differentiation in the amygdala and hippocampus [177, 178] In terms of neuromodulation, 5 Hz repetitive TMS (rTMS) improved chronic stress-induced myelin damage and depressive-like behaviors by increasing the number of Olig2^+^ OLs in the PFC and hippocampus [179]. This effect was independent of the neurogenesis-promotion function of fluoxetine and showed synergistic effects in combination therapy [179].

Metabolic interventions based on gut-brain axis shows unique potential. Enterococcus faecalis EF-2001 reversed depressive-like behaviors in olfactory bulbectomy mice model by activating the neuronal CREB/BDNF and astrocyte-based LIF/STAT3 signaling pathways to promote OPCs differentiation and myelination in the PFC [180]. Dietary metabolic studies reveal that a high-fat diet rich in palmitic acid can partially compensate for fatty acid synthesis defects in OLs, improving developmental myelin sheath growth, suggesting the potential value of dietary lipid intake for myelin repairment [181]. Additionally, the ketogenic diet (KD), as a non-pharmacological treatment for pediatric refractory epilepsy, has demonstrated myelin repairment effects in numerous preclinical and clinical studies related to multiple sclerosis, with increasing evidence supporting its antidepressant potential [182–186]. Leclercq S et al. [187] showed that gut microbiota dysbiosis-induced inhibition of β-hydroxybutyrate synthesis was associated with social impairment, depression, and white matter alterations in alcohol use disorder, implying that the KD may exert antidepressant effects through OLs-targeted mechanisms. Omega-3 fatty acids (ω-3), particularly eicosapentaenoic acid and docosahexaenoic acid, showed adjuvant antidepressant efficacy by reducing sleep deprivation-induced myelin damage and OLs lipid peroxidation [188, 189]. It also rescued offspring from depressive behaviors caused by maternal high-fat diet during gestation and lactation period via improving the suppression of MOG and myelin and lymphocyte protein expression, restoring the reduced number of OPCs and mature OLs in the PFC and cingulate cortex [190]. Notably, there was sex-specific heterogeneity of effect of ω-3 on depression: significant in male offspring but not in female rats [190].

Exercise-induced antidepressant effects are also related to OL lineage cells function and myelin remodeling. A DTI study found that mice exhibited increased hippocampus volume, with FA values positively correlated with exercise intensity, along with an increased number of OPCs in the corpus callosum [191]. Consistently, running exercise protected white matter integrity in CUMS-induced depression rats by restoring myelinated fiber length, myelin sheath volume and thickness, thereby rescuing behavioral deficits [192]. In a chronic stress model, running exercise (but not fluoxetine) specifically increased the number of mature OLs (CC1^+^/Olig2^+^) and MBP levels in the CA1 region, while promoting OPCs proliferation in the PFC [193]. Further evidence revealed that treadmill running alleviated depressive-like behaviors while increasing the number of CNPase^+^ mature OLs in hippocampal CA3 and dentate gyrus, highlighting a reginal heterogeneity of exercise-induced OLs protection [194]. This effect showed developmental stage dependency: adolescent exercise increased the number of mature OLs in the PFC, whereas young adult exercise had more significant effects on inducing OPCs differentiation [195]. Long-term exercise promoted increased MCT1 in the PFC, suggesting that exercise might exert antidepressant effects by rescuing neuronal energy deficits through OLs-mediated lactate shuttle [195].

In summary, non-pharmacological strategies targeting OL lineage cells (physical intervention, metabolic intervention, and lifestyle improvement) provide a multidimensional intervention for the treatment of depression by regulating myelination, OPCs proliferation, and OL-neuronal interactions. Collectively, these findings highlight OL lineage cells as promising therapeutic targets, while future studies should clarify the translational efficacy, developmental stage specificity, and long-term safety of these pharmacological and non-pharmacological interventions in depression.

## Conclusions

The pathogenesis of depression remains incompletely understood. Compared to adult depression, adolescent depression has a more insidious onset, complicating diagnosis and limiting treatment options - implying a potentially distinct underlying pathology. OLs, once considered merely as myelin-producing cells, are now recognized for their non-myelinating roles, including energy provision, metabolic regulation, immune response, and modulation of synaptic plasticity. Notably, emerging evidence shows that OPCs interact with neurons via synaptic contacts and participate in synapse modulation during development, a process further shaped by microglia [17, 61]. These findings suggest that OL lineage cells may contribute to neuro-immune-metabolic interactions and their potential involvement in the pathology of adolescent depression. However, most studies still focus on myelin function, with limited exploration of OL lineage cells’ non-canonical roles. Moreover, findings on the spatial, temporal, and sex-specific heterogeneity of OL lineage cells in depression remain inconsistent. Current research is largely based on adult models, lacking dynamic developmental-stage tracking, which restricts our understanding of adolescent-specific pathology. Additionally, validated biomarkers associated with OL lineage cells along with non-invasive tools for in vivo assessment of OL-specific pathology are still lacking, limiting the translational potential of OL-targeted research. Advances in neuroimaging techniques, such as high-resolution magnetic resonance spectroscopy and positron emission tomography (PET) molecular probes, have shown promise in tracking neurotransmission in clinical depression cohorts [196–198]. However, most of these approaches are not designed to specifically capture oligodendrocyte dynamics, especially in developmental populations.

To address these challenges, future research can be expanded in the following directions. First, studies based on single-cell spatial omics could uncover transcriptional, lipid metabolic, and epigenetic characteristics of OL lineage cells across brain regions, identifying potential pathological subtypes and key regulatory molecules. Second, studies should focus on the developmental time windows and causality validation, especially regarding OPCs-neuron communication, to dissect how OL lineage cells regulate neural plasticity through metabolic coupling and immune crosstalk with neurons, microglia, and astrocytes. Third, based on the evidence that ELS exerts persistent and multifaceted impacts on OL lineage cells, future studies should also aim to disentangle how ELS-induced alternations in OPC differentiation, myelination dynamics, and metabolic programming affect the maturation of neural circuits and constrain synaptic plasticity, thereby heightening vulnerability to adolescent depression. Longitudinal studies integrating developmental tracking with mechanistic validation in both animal models and human cohorts are needed to identify critical windows of susceptibility and clarify the causal pathways. Given the emerging evidence linking lipid metabolism to oligodendrocyte development, myelin integrity, and neural plasticity, future studies should systematically explore lipid-derived biomarkers and lipid-targeted interventions. Finally, future translational research should prioritize the development of OL-targeted imaging probes (e.g., PET ligands for myelin-associated proteins or lipid metabolism markers). Efforts should be directed toward identifying adolescent-relevant OL lineage cells derived biomarkers, developing precise interventions based on mechanistic insights, and evaluating the developmental-stage-specific efficacy of existing treatments, including small molecule antidepressants, metabolic modulators, and physical therapies, to advance precision treatment of adolescent depression.
